# Post-operative transmesosigmoid hernia causing small bowel obstruction: a case report

**DOI:** 10.11604/pamj.2015.20.318.5752

**Published:** 2015-04-01

**Authors:** Robleh Hassan Farah, Yassine Fahmi, Driss Khaiz, Khalid Elhattabi, Fatimazahra Bensardi, Rachid Lefriyekh, Saad Berrada, Abdelaziz Fadil, Najib Zerouali Ouariti

**Affiliations:** 1 Service des Urgences Chirurgicales Viscérales, Centre Hospitalier Universitaire Ibn Rochd, Casablanca, Morocco

**Keywords:** Internal hernia, transmesosigmoid hernia, intestinal obstruction

## Abstract

Internal hernia is an unusual cause of small bowel obstruction and classified several types according to locations. Transmesosigmoid hernia is rare type among others mesosigmoid hernia was rarely reported in the literature. We report the case of a 44-year-old male who presented with acute abdominal pain and developed a small intestinal obstruction. History, clinical and radiography examination were suggested intestinal obstruction due to postoperative adhesion. The diagnosis of small bowel obstruction due to internal hernia was confirmed by laparotomy exploration. The herniated loop was reduced successfully and the defect was approximated with interrupted sutures. The postoperative course was uneventful and the patient is free from symptoms and recurrence. This case report highlight difficulty and importance of high index of suspicion considering an internal hernia as a cause of small bowel obstruction in individuals of all age groups with or without a previous history of abdominal surgery.

## Introduction

Internal hernia is the protrusion of a viscus through a normal or abnormal mesenteric or peritoneal aperture. A study of more than 20,000 cases of intestinal obstruction in Japan has shown the incidence of internal hernia to be only 0.7% [1]. It causes up to 5.8% of cases of small bowel obstruction (SBO). The common internal hernias include paraduodenal, transomental, mesenteric defects and through foramen of Winslow but Sigmoid related hernias are especially rare, and account for only 6% of internal hernias [2].

## Patient and observation

**Figure 1:**
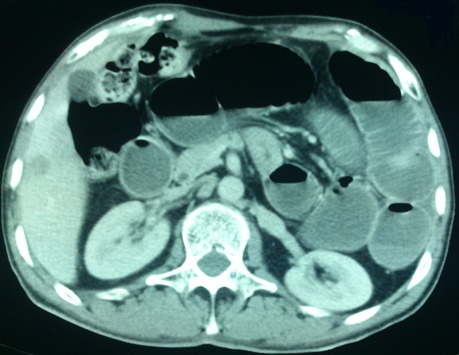
Abdominal CT scan demonstrating small intestinal dilatation with free air fluid level

**Figure 2:**
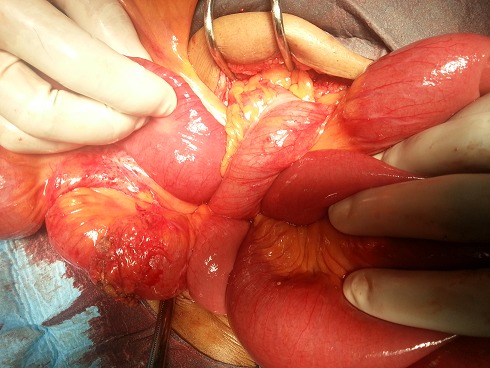
Intra operative findings of herniated small bowel through the sigmoid mesocolon (transmesosigmoid)

A 44-year-old man presented acutely with a 1-day history of colicky central abdominal pain, abdominal distension, vomiting and absolute constipation. He had previous history of left segmental colectomy 4 months ago. Physical examination revealed dry mucous membranes and tenderness in the left lower quadrant. Bowel sounds were increased and digital rectal examination empty rectal pouch. Laboratory investigations revealed a leukocytosis of 12400/mm^3^. Plain abdominal radiography demonstrated two prominent loops of small bowel in the left lower quadrant. Nasogastric tube was inserted and he was resuscitated with intravenous fluids. A computed tomography (CT) scan of abdomen showed small intestinal obstruction caused by post-operative adhesion (Figure 1) with dilated loops of small bowel. Based on his clinical and CT findings a decision was made to perform an exploratory laparotomy. At laparotomy, a mechanical small bowel obstruction due to an incarcerated internal hernia was found. A loop of ileum had herniated through a post-operative defect in the mesosigmoid (Figure 2). The herniated loop was reduced successfully and the defect was approximated with interrupted 3/ 0 poliglecaprone sutures. The strangulated portion of small bowel was found to be viable. In this case the hernial orifice measured 4.5 cm and consisted of two leaves of the sigmoid mesentery. No hernial sac was present.

## Discussion

Internal hernia is defined as a protrusion of an organ, usually the small intestine, through acquired or congenital aperture within the abdominal cavity. The incidence of internal hernia is estimated to account for approximately 1-6% of intestinal obstruction [3]. Sigmoid mesocolon hernia accounts only for 6% of all internal hernias [3–5]. Benson and Killen and Bircher and Stuart reported that there are three subtypes of sigmoid hernia; namely, intersigmoid hernia, which arises in the congenital fossa situated in the attachment of the lateral aspect of the sigmoid mesocolon to the posterior abdominal wall; transmesosigmoid hernia, which occurs when loops of intestine pass through a defect in the sigmoid mesocolon (no actual hernia sac is present in this condition); and intermesosigmoid hernia, which occurs when the defect in the sigmoid mesocolon affects only the left leaf of the peritoneum, and the hernial sac lies within the sigmoid mesocolon itself. Sigmoid hernias account for about 5% of all internal hernias [6]. In our case report the mesosigmoid hernia was of transmesosigmoid type (Figure 1). Transmesosigmoid hernia no sex predominance, but some case reports have documented of transmesosigmoid hernias developing during pregnancy or postpartum. The authors of these reports proposed that dilatation and shrinkage of the uterus concomitant with pregnancy or delivery contributed to the development of transmesosigmoid hernias & usually associated with a significantly long intestinal loop herniated into the opposite side of the mesocolon, perhaps because of a trend toward protrusion of the intestine [7]. The exact nature of defects in the mesentery remains a mystery, both acquired and congenital etiologies have been proposed. Acquired risk factors include trauma, previous surgery laparoscopic or laparotomy and intra-abdominal inflammation [8].

In our case had previous segmental left colectomy due to left colon polyp. In cases of transmesosigmoid hernias, patients tend to present acutely with abdominal pain and signs of small bowel obstruction [9]. However, making a pre-operative diagnosis of an internal hernia, even with recent Radiological investigations such as CT scan of the abdomen can be difficult to establish certain diagnose, and therefore the majority of cases are confirmed per-operatively. The management of internal hernias requires reduction of the hernia and repair of the defect by either a laparoscopic or open approach. In these cases there is a high incidence of small bowel ischaemia, infarction and resection of the strangulated small bowel segment may be necessary. Iatrogenic internal hernias can be successfully managed by laparoscopy and laparoscopic repair of congenital internal hernia has been described [10]. In our case, an emergency laparotomy was performed based on clinical examination and abdominal CT findings with preoperative provisional diagnosis of adhesive small bowel obstruction. If an internal herniation is suspected, the surgery should be prompt, as strangulation and necrosis of the hernial contents is likely to ensue if the surgery is delayed [11]. In the majority of cases, partial resection of small intestine is necessary. Recently, some authors have recommended laparoscopic abdominal surgery for both diagnosis and treatment. The results of surgical treatment for sigmoid mesocolon hernias are good [5, 7].

## Conclusion

Although, transmesosigmoid hernia is a cause rare of small bowel obstruction. It should be considered in the differential diagnosis in patients with suspected incarceration or strangulation. It is important to make decision of timely surgical intervention to reduce morbidity and mortality of the disease.
